# Supplementary material for: Analysis of PPARγ Signaling Activity in Psoriasis
Source: Int J Mol Sci. 2021 Aug 10;22(16):8603. doi: 10.3390/ijms22168603 (PMC8395241; doi:10.3390/ijms22168603)
Supplement: Supplementary file 1 [file ijms-22-08603-s001.zip › Supplemental materials_Analysis of PPARg signaling activity in psoriasis/Pathway models/Models images and html files/PPARG expressed in cells assotiated with psoriasis/225520.html]

natural killer cell


# CellType natural killer cell

|  |  |
| --- | --- |
| URN | urn:agi-ncimcelltype:C0022688 |
| Total Entities | 65 |
| Connectivity | 5543 |
| Name | natural killer cell |
| ObjectType | Cell |

---

|  |  |
| --- | --- |
| ChildConcepts | natural killer cell differentiation |
|  | natural killer cell proliferation |
|  | NK cell mediated cytotoxicity |
|  | natural killer cell chemotaxis |
|  | natural killer cell apoptosis |
|  | CD56+ natural killer cell |
|  | CD56hi natural killer cell |
|  | CD8+ natural killer cell |
|  | CD57+ natural killer cell |
|  | activated CD56+ natural killer cell |
|  | CD16+ CD57+ natural killer cell |
|  | activated natural killer cell |
|  | NK1.1+ natural killer cell |
|  | CD56low natural killer cell |
|  | CD16- CD56hi natural killer cell |
|  | decidual natural killer cell |
|  | natural killer cell development |
|  | natural killer cell migration |
|  | natural killer cell survival |
|  | natural killer cell phenotype |
|  | natural killer cell fate |
|  | natural killer cell homeostasis |
|  | natural killer cell recognition |
|  | natural killer cell population |
|  | natural killer cell infiltration |
|  | natural killer cell formation |
|  | natural killer cell motility |
|  | natural killer cell adhesion |
|  | natural killer cell distribution |
|  | natural killer cell death |
|  | natural killer cell interaction |
|  | natural killer cell rolling |
|  | natural killer cell growth |
|  | natural killer cell division |
|  | natural killer cell sequestration |
|  | natural killer cell homing |
|  | natural killer cell extravasation |
|  | natural killer cell polarity |
|  | natural killer cell viability |
|  | natural killer cell proliferative response |
|  | natural killer cell priming |
|  | natural killer cell invasion |
|  | natural killer cell neutrophil chemotaxis |
|  | natural killer cell repertoire |
|  | natural killer cell contact |
|  | natural killer cell destruction |
|  | natural killer cell transendothelial migration |
|  | natural killer cell regeneration |
|  | natural killer cell ratio |
|  | natural killer cell aggregation |
|  | natural killer cell fate commitment |
|  | natural killer cell damage |
|  | natural killer cell localization |
|  | natural killer cell developmental process |
|  | natural killer cell phagocytosis |
|  | natural killer cell size |
|  | natural killer cell renewal |
|  | natural killer cell senescence |
|  | natural killer cell selection |
|  | natural killer cell clonal anergy |
|  | natural killer cell motion |
|  | natural killer cell autophagy |
|  | natural killer cell spreading |
|  | natural killer cell stress |
|  | natural killer cell tethering |

---

|  |  |
| --- | --- |
| Pathway | Natural Killer Cell Precursor -> Natural Killer Cell Surface Expression Markers |
|  | IL12 Antitumor Effect is Blocked in Cancer |
|  | Myeloid Derived Suppressor Cells in Cancer Immune Escape |
|  | IDO1 in Cancer Immune Escape |
|  | PPARG expressed in cells assotiated with psoriasis |
|  | Vg9Vd2 cells-\|SARS |
|  | 5\_Tax-mediated dysregulation of NF-kB signaling in HTLV-1 infection |
|  | 3\_Islet beta-cell destruction in T1D |

---

|  |  |
| --- | --- |
| MedScan ID | 10003430 |

---

|  |  |
| --- | --- |
| Alias | natural killer (NK)-cell |
|  | NK cells |
|  | N-K cells |
|  | N-K cell |
|  | natural killer (NK)-cells |
|  | natural killer cells |
|  | NK cell |
|  | cell strain NK |
|  | natural killer cell |

---

|  |  |
| --- | --- |
| NCIm ID | C0022688 |

---
